# 1-Meth­oxy-2-methyl-1*H*-benzo[*f*]indole-3-carbonitrile

**DOI:** 10.1107/S1600536809052416

**Published:** 2009-12-09

**Authors:** Jiang-Sheng Li, Qi-Xi He, Peng-Yu Li

**Affiliations:** a School of Chemistry and Biological Engineering, Changsha University of Science & Technology, Changsha 410004, People’s Republic of China; b College of Chemistry and Chemical Engineering, Hunan University, Changsha 410082, People’s Republic of China

## Abstract

Apart from the methyl group of the meth­oxy fragment, the title compound, C_15_H_12_N_2_O, is almost planar (r.m.s. deviation = 0.045 Å); the C atom deviates from the mean plane by 1.216 (1) Å. In the crystal, π–π stacking [shortest centroid–centroid separation = 3.4652 (10) Å] and C—H⋯π inter­actions occur.

## Related literature

For the synthesis, see: Du *et al.* (2008 ▶).
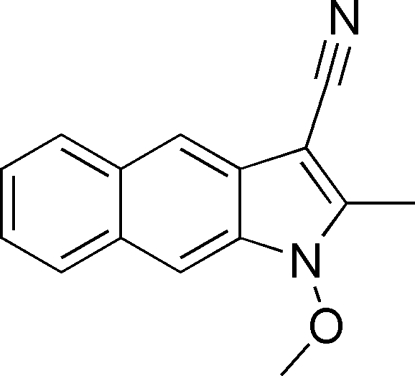


## Experimental

### 

#### Crystal data

C_15_H_12_N_2_O
*M* *_r_* = 236.27
Monoclinic, 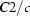

*a* = 18.663 (4) Å
*b* = 7.3763 (15) Å
*c* = 18.589 (4) Å
β = 113.46 (3)°
*V* = 2347.6 (8) Å^3^
*Z* = 8
Mo *K*α radiation
μ = 0.09 mm^−1^
*T* = 113 K
0.20 × 0.18 × 0.16 mm

#### Data collection

Rigaku Saturn CCD diffractometer
Absorption correction: multi-scan (*CrystalClear*; Rigaku/MSC, 2005 ▶) *T*_min_ = 0.983, *T*_max_ = 0.986
11412 measured reflections
2060 independent reflections
1858 reflections with *I* > 2σ(*I*)
*R*_int_ = 0.035

#### Refinement

*R*[*F* ^2^ > 2σ(*F* ^2^)] = 0.035
*wR*(*F* ^2^) = 0.094
*S* = 1.04
2060 reflections
166 parameters
H-atom parameters constrained
Δρ_max_ = 0.19 e Å^−3^
Δρ_min_ = −0.16 e Å^−3^

### 

Data collection: *CrystalClear* (Rigaku/MSC, 2005 ▶); cell refinement: *CrystalClear*; data reduction: *CrystalClear*; program(s) used to solve structure: *SHELXS97* (Sheldrick, 2008 ▶); program(s) used to refine structure: *SHELXL97* (Sheldrick, 2008 ▶); molecular graphics: *SHELXTL* (Sheldrick, 2008 ▶); software used to prepare material for publication: *SHELXL97*.

## Supplementary Material

Crystal structure: contains datablocks I, global. DOI: 10.1107/S1600536809052416/hb5274sup1.cif

Structure factors: contains datablocks I. DOI: 10.1107/S1600536809052416/hb5274Isup2.hkl

Additional supplementary materials:  crystallographic information; 3D view; checkCIF report

## Figures and Tables

**Table 1:** Hydrogen-bond geometry (Å, °) *Cg*3 is the centroid of the C4–C9 ring.

*D*—H⋯*A*	*D*—H	H⋯*A*	*D*⋯*A*	*D*—H⋯*A*
C3—H3⋯*Cg*3^i^	0.93	2.65	3.3956 (15)	138

Symmetry code: (i) 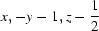.

**Fig. 1.**
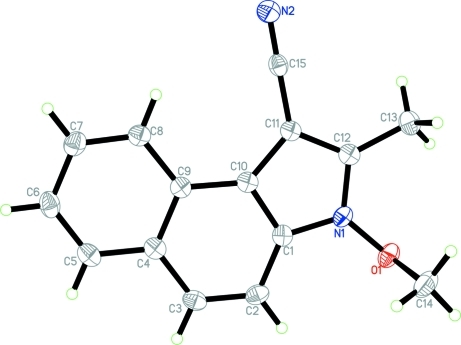
The molecular structure of (I) showing displacement ellipsoids drawn at the 50% probability level.

## supplementary crystallographic information

### Comment

The title compound, (I), comprises of a benzo ring and its fused indole ring (Fig. 1). The aromatic skeleton is essentially planar.

In the crystal packing, π-π stacking interaction and C—H···π interaction help establish the molecular packing. The shortest centroid-centroid separation is 3.4652 (10) Å, which occurs between the pyrrole parts of the molecules.

### Experimental

The compound was obtained according to the method of Du and his coworkers (2008). Colourless block of (I) was grown by slow evaporation of its ethanolic solution.

### Refinement

All H atoms were positioned geometrically (C—H = 0.93 and 0.96 Å)and refined as riding with *U*_iso_(H) = 1.2*U*_eq_(CH) or 1.5*U*_eq_(CH_3_).

### Crystal data

**Table:**

C_15_H_12_N_2_O	*F*(000) = 992
*M**_r_* = 236.27	*D*_x_ = 1.337 Mg m^−^^3^
Monoclinic, *C*2/*c*	Mo *K*α radiation, λ = 0.71073 Å
Hall symbol: -C 2yc	Cell parameters from 3553 reflections
*a* = 18.663 (4) Å	θ = 2.2–27.9°
*b* = 7.3763 (15) Å	µ = 0.09 mm^−^^1^
*c* = 18.589 (4) Å	*T* = 113 K
β = 113.46 (3)°	Block, colourless
*V* = 2347.6 (8) Å^3^	0.20 × 0.18 × 0.16 mm
*Z* = 8	

### Data collection

**Table:**

Rigaku Saturn CCD diffractometer	2060 independent reflections
Radiation source: rotating anode	1858 reflections with *I* > 2σ(*I*)
confocal	*R*_int_ = 0.035
Detector resolution: 7.31 pixels mm^-1^	θ_max_ = 25.0°, θ_min_ = 2.4°
ω and φ scans	*h* = −22→22
Absorption correction: multi-scan (*CrystalClear*; Rigaku/MSC, 2005)	*k* = −8→8
*T*_min_ = 0.983, *T*_max_ = 0.986	*l* = −22→22
11412 measured reflections	

### Refinement

**Table:**

Refinement on *F*^2^	Secondary atom site location: difference Fourier map
Least-squares matrix: full	Hydrogen site location: inferred from neighbouring sites
*R*[*F*^2^ > 2σ(*F*^2^)] = 0.035	H-atom parameters constrained
*wR*(*F*^2^) = 0.094	*w* = 1/[σ^2^(*F*_o_^2^) + (0.0542*P*)^2^ + 1.1054*P*] where *P* = (*F*_o_^2^ + 2*F*_c_^2^)/3
*S* = 1.04	(Δ/σ)_max_ < 0.001
2060 reflections	Δρ_max_ = 0.19 e Å^−^^3^
166 parameters	Δρ_min_ = −0.16 e Å^−^^3^
0 restraints	Extinction correction: *SHELXL97* (Sheldrick, 2008), Fc^*^=kFc[1+0.001xFc^2^λ^3^/sin(2θ)]^-1/4^
Primary atom site location: structure-invariant direct methods	Extinction coefficient: 0.0212 (18)

### Special details

**Table:**

Geometry. All e.s.d.'s (except the e.s.d. in the dihedral angle between two l.s. planes) are estimated using the full covariance matrix. The cell e.s.d.'s are taken into account individually in the estimation of e.s.d.'s in distances, angles and torsion angles; correlations between e.s.d.'s in cell parameters are only used when they are defined by crystal symmetry. An approximate (isotropic) treatment of cell e.s.d.'s is used for estimating e.s.d.'s involving l.s. planes.
Refinement. Refinement of *F*^2^ against ALL reflections. The weighted *R*-factor *wR* and goodness of fit *S* are based on *F*^2^, conventional *R*-factors *R* are based on *F*, with *F* set to zero for negative *F*^2^. The threshold expression of *F*^2^ > σ(*F*^2^) is used only for calculating *R*-factors(gt) *etc*. and is not relevant to the choice of reflections for refinement. *R*-factors based on *F*^2^ are statistically about twice as large as those based on *F*, and *R*- factors based on ALL data will be even larger.

### Fractional atomic coordinates and isotropic or equivalent isotropic displacement parameters (Å^2^)

**Table:**

	*x*	*y*	*z*	*U*_iso_*/*U*_eq_	
O1	0.43321 (5)	0.84231 (12)	0.08385 (5)	0.0257 (3)	
N1	0.35813 (6)	0.90176 (14)	0.06827 (6)	0.0203 (3)	
N2	0.11930 (6)	1.14000 (15)	−0.10753 (6)	0.0269 (3)	
C1	0.32134 (7)	0.87498 (16)	0.11865 (7)	0.0194 (3)	
C2	0.34992 (7)	0.79261 (17)	0.19296 (7)	0.0223 (3)	
H2	0.4010	0.7503	0.2163	0.027*	
C3	0.29960 (7)	0.77742 (16)	0.22936 (7)	0.0223 (3)	
H3	0.3171	0.7248	0.2789	0.027*	
C4	0.22060 (7)	0.83997 (16)	0.19371 (7)	0.0201 (3)	
C5	0.16852 (7)	0.81573 (17)	0.23131 (7)	0.0235 (3)	
H5	0.1861	0.7589	0.2800	0.028*	
C6	0.09292 (7)	0.87424 (18)	0.19742 (7)	0.0256 (3)	
H6	0.0597	0.8579	0.2232	0.031*	
C7	0.06515 (8)	0.95940 (18)	0.12338 (7)	0.0249 (3)	
H7	0.0138	1.0002	0.1008	0.030*	
C8	0.11331 (7)	0.98234 (16)	0.08458 (7)	0.0208 (3)	
H8	0.0941	1.0372	0.0355	0.025*	
C9	0.19198 (7)	0.92382 (15)	0.11816 (7)	0.0181 (3)	
C10	0.24518 (7)	0.93956 (15)	0.08037 (7)	0.0181 (3)	
C11	0.23885 (7)	1.00368 (15)	0.00486 (7)	0.0185 (3)	
C12	0.31010 (7)	0.97803 (16)	−0.00040 (7)	0.0195 (3)	
C13	0.33588 (8)	1.02295 (18)	−0.06433 (7)	0.0253 (3)	
H13A	0.3631	1.1367	−0.0531	0.038*	
H13B	0.2911	1.0317	−0.1131	0.038*	
H13C	0.3700	0.9295	−0.0681	0.038*	
C14	0.48901 (8)	0.9831 (2)	0.12264 (9)	0.0327 (4)	
H14A	0.4818	1.0829	0.0873	0.049*	
H14B	0.5410	0.9362	0.1383	0.049*	
H14C	0.4813	1.0239	0.1681	0.049*	
C15	0.17262 (7)	1.07923 (16)	−0.05700 (7)	0.0196 (3)	

### Atomic displacement parameters (Å^2^)

**Table:**

	*U*^11^	*U*^22^	*U*^33^	*U*^12^	*U*^13^	*U*^23^
O1	0.0194 (5)	0.0244 (5)	0.0344 (5)	0.0039 (4)	0.0119 (4)	−0.0008 (4)
N1	0.0163 (5)	0.0211 (5)	0.0235 (6)	0.0016 (4)	0.0079 (4)	−0.0004 (4)
N2	0.0258 (6)	0.0317 (7)	0.0227 (6)	0.0015 (5)	0.0093 (5)	0.0016 (5)
C1	0.0211 (6)	0.0161 (6)	0.0216 (6)	−0.0016 (5)	0.0090 (5)	−0.0021 (5)
C2	0.0203 (6)	0.0193 (6)	0.0237 (7)	0.0015 (5)	0.0049 (5)	−0.0005 (5)
C3	0.0274 (7)	0.0180 (6)	0.0190 (6)	−0.0013 (5)	0.0067 (5)	0.0008 (5)
C4	0.0241 (7)	0.0155 (6)	0.0201 (6)	−0.0031 (5)	0.0082 (5)	−0.0034 (5)
C5	0.0300 (7)	0.0205 (6)	0.0200 (6)	−0.0063 (5)	0.0100 (5)	−0.0031 (5)
C6	0.0265 (7)	0.0283 (7)	0.0258 (7)	−0.0094 (6)	0.0145 (6)	−0.0062 (5)
C7	0.0199 (6)	0.0273 (7)	0.0259 (7)	−0.0043 (5)	0.0076 (5)	−0.0063 (5)
C8	0.0209 (6)	0.0200 (6)	0.0190 (6)	−0.0034 (5)	0.0054 (5)	−0.0030 (5)
C9	0.0203 (6)	0.0142 (6)	0.0187 (6)	−0.0042 (5)	0.0067 (5)	−0.0044 (5)
C10	0.0208 (6)	0.0135 (6)	0.0188 (6)	−0.0030 (5)	0.0065 (5)	−0.0033 (5)
C11	0.0203 (6)	0.0158 (6)	0.0181 (6)	−0.0023 (5)	0.0064 (5)	−0.0019 (4)
C12	0.0227 (7)	0.0153 (6)	0.0198 (6)	−0.0026 (5)	0.0076 (5)	−0.0032 (5)
C13	0.0295 (7)	0.0232 (7)	0.0262 (7)	−0.0014 (5)	0.0143 (6)	−0.0012 (5)
C14	0.0183 (7)	0.0371 (8)	0.0394 (8)	−0.0031 (6)	0.0079 (6)	−0.0001 (6)
C15	0.0225 (7)	0.0191 (6)	0.0192 (6)	−0.0034 (5)	0.0104 (5)	−0.0025 (5)

### Geometric parameters (Å, °)

**Table:**

O1—N1	1.3844 (13)	C6—H6	0.9300
O1—C14	1.4442 (16)	C7—C8	1.3685 (18)
N1—C12	1.3559 (16)	C7—H7	0.9300
N1—C1	1.3784 (16)	C8—C9	1.4149 (18)
N2—C15	1.1529 (16)	C8—H8	0.9300
C1—C10	1.3958 (17)	C9—C10	1.4312 (17)
C1—C2	1.4054 (17)	C10—C11	1.4408 (16)
C2—C3	1.3631 (19)	C11—C12	1.3846 (18)
C2—H2	0.9300	C11—C15	1.4245 (18)
C3—C4	1.4311 (18)	C12—C13	1.4862 (17)
C3—H3	0.9300	C13—H13A	0.9600
C4—C5	1.4163 (18)	C13—H13B	0.9600
C4—C9	1.4292 (17)	C13—H13C	0.9600
C5—C6	1.3662 (19)	C14—H14A	0.9600
C5—H5	0.9300	C14—H14B	0.9600
C6—C7	1.4106 (19)	C14—H14C	0.9600
			
N1—O1—C14	110.19 (9)	C9—C8—H8	119.5
C12—N1—C1	112.19 (10)	C8—C9—C4	118.80 (11)
C12—N1—O1	124.28 (10)	C8—C9—C10	124.02 (11)
C1—N1—O1	123.30 (10)	C4—C9—C10	117.16 (11)
N1—C1—C10	106.64 (11)	C1—C10—C9	119.12 (11)
N1—C1—C2	129.27 (11)	C1—C10—C11	106.30 (11)
C10—C1—C2	124.03 (12)	C9—C10—C11	134.51 (11)
C3—C2—C1	117.16 (11)	C12—C11—C15	123.07 (11)
C3—C2—H2	121.4	C12—C11—C10	108.38 (11)
C1—C2—H2	121.4	C15—C11—C10	128.54 (11)
C2—C3—C4	122.01 (12)	N1—C12—C11	106.49 (11)
C2—C3—H3	119.0	N1—C12—C13	122.83 (11)
C4—C3—H3	119.0	C11—C12—C13	130.67 (12)
C5—C4—C9	118.56 (11)	C12—C13—H13A	109.5
C5—C4—C3	120.91 (11)	C12—C13—H13B	109.5
C9—C4—C3	120.51 (11)	H13A—C13—H13B	109.5
C6—C5—C4	121.21 (12)	C12—C13—H13C	109.5
C6—C5—H5	119.4	H13A—C13—H13C	109.5
C4—C5—H5	119.4	H13B—C13—H13C	109.5
C5—C6—C7	120.10 (12)	O1—C14—H14A	109.5
C5—C6—H6	120.0	O1—C14—H14B	109.5
C7—C6—H6	120.0	H14A—C14—H14B	109.5
C8—C7—C6	120.40 (12)	O1—C14—H14C	109.5
C8—C7—H7	119.8	H14A—C14—H14C	109.5
C6—C7—H7	119.8	H14B—C14—H14C	109.5
C7—C8—C9	120.91 (12)	N2—C15—C11	179.39 (13)
C7—C8—H8	119.5		
			
C14—O1—N1—C12	93.87 (13)	N1—C1—C10—C9	178.00 (10)
C14—O1—N1—C1	−92.15 (13)	C2—C1—C10—C9	0.53 (18)
C12—N1—C1—C10	−0.59 (13)	N1—C1—C10—C11	0.58 (12)
O1—N1—C1—C10	−175.22 (10)	C2—C1—C10—C11	−176.89 (11)
C12—N1—C1—C2	176.70 (12)	C8—C9—C10—C1	−179.17 (11)
O1—N1—C1—C2	2.08 (19)	C4—C9—C10—C1	−0.57 (16)
N1—C1—C2—C3	−177.49 (11)	C8—C9—C10—C11	−2.7 (2)
C10—C1—C2—C3	−0.62 (18)	C4—C9—C10—C11	175.95 (12)
C1—C2—C3—C4	0.78 (18)	C1—C10—C11—C12	−0.40 (13)
C2—C3—C4—C5	177.32 (11)	C9—C10—C11—C12	−177.24 (12)
C2—C3—C4—C9	−0.89 (18)	C1—C10—C11—C15	178.68 (11)
C9—C4—C5—C6	−1.41 (18)	C9—C10—C11—C15	1.8 (2)
C3—C4—C5—C6	−179.65 (11)	C1—N1—C12—C11	0.33 (13)
C4—C5—C6—C7	0.48 (19)	O1—N1—C12—C11	174.90 (10)
C5—C6—C7—C8	0.70 (19)	C1—N1—C12—C13	179.24 (11)
C6—C7—C8—C9	−0.91 (18)	O1—N1—C12—C13	−6.20 (18)
C7—C8—C9—C4	−0.04 (17)	C15—C11—C12—N1	−179.09 (10)
C7—C8—C9—C10	178.54 (11)	C10—C11—C12—N1	0.05 (13)
C5—C4—C9—C8	1.18 (17)	C15—C11—C12—C13	2.1 (2)
C3—C4—C9—C8	179.42 (10)	C10—C11—C12—C13	−178.73 (12)
C5—C4—C9—C10	−177.50 (10)	C12—C11—C15—N2	20 (14)
C3—C4—C9—C10	0.75 (17)	C10—C11—C15—N2	−159 (14)

### Hydrogen-bond geometry (Å, °)

**Table:**

Cg3 is the centroid of the C4–C9 ring.

**Table:**

*D*—H···*A*	*D*—H	H···*A*	*D*···*A*	*D*—H···*A*
C3—H3···Cg3^i^	0.93	2.65	3.3956 (15)	138

Symmetry codes: (i) *x*, −*y*−1, *z*−1/2.
